# Evaluation of eyebrow position and upper eyelid laxity after endolift laser treatment

**DOI:** 10.1111/srt.13498

**Published:** 2023-10-12

**Authors:** Elaheh Lotfi, Roberto DellAvanzato, Najmeh Ahramiyanpour, Fatemeh Shadmanyazdi, Somayeh Khosravi

**Affiliations:** ^1^ Yousef Abad Skin and Hair Center Tehran Iran; ^2^ Ambers Laserklinik Gothenburg Sweden; ^3^ La Madonnina Clinic Milano Italy; ^4^ Department of Dermatology Afzalipour faculty of Medicine Afzalipour Hospital Kerman University of Medical Sciences Kerman Iran; ^5^ Imam Khomeini Hospital Complex Advanced Medical Technologies & Equipment Institute Tehran Iran

**Keywords:** endolift laser, eyebrow position, treatment, upper eyelid laxity

## Abstract

The periorbital area is one of the most sensitive areas in the face, and many techniques are used to change the eyelid laxity and position of the eyebrows. Recently the use of noninvasive or minimally invasive techniques is developed so, the amount of surgical procedures has decreased. In the present study. We evaluate the effect of Endolift laser as a non‐invasive method on upper eyelid laxity and eyebrow position. Nine patients underwent Endolift laser and evaluated for eyelid laxity and change in eyebrow position. Two blinded physicians assessed the cosmetic outcome of improvement in upper eyelid tightening via photography 6 months post‐treatment. The physicians evaluated the patient's improvement degree using the photographs by two clinical factors: skin laxity and total cosmetic result. Also, eyebrow height from the center of the pupil (CPEBH), Central eyebrow height (CEBH), Lateral eyebrow height (LEBH), Medial eyebrow height (MEBH) were measured by ImagJ before and 6 months post Endolift laser treatment. Our results showed eyelid laxity and eyebrow height were significantly changed after treatment.

## 1 INTRODUCTION

Aging causes the appearance of extra skin nearby the eyelids with increasing skin laxity and eyebrow ptosis so this is a common disorder that many patients suffer from. Improvement of eyelid skin texture, excessive eyelid skin laxity and tightening of eyelid skin, and increase height of eyebrows are the common reasons that people visit a dermatologist. Eyelid skin tightening and excessive eyelid skin laxity correction can be done in a variety of methods such as dermal filler and chemical peels injections and laser therapy besides multiple plastic surgery methods.^1^, ^2^, ^3^ Because of specific skin structure the eye area is one of the most difficult regions to use aesthetic techniques.^4^ Eyebrow lift and blepharoplasty are surgical techniques that are commonly used for the treatment of periorbital aging but it is accompanied by pain and need long recovery time and possible side effects.^4^ Several nonsurgical treatments are studied as an alternative to surgical blepharoplasties, like, erbium‐YAG laser, ablative CO2 and fractional ablative CO2 laser and non‐ablative fractional laser, soft tissue fillers, radio‐frequency technology.^3^, ^5^ Some of these methods may be combined to get better results. Each technique has its weaknesses and strengths. To evade the significant potential side effects of ablative techniques, the non‐ablative methods are advanced. Generally, in the field of skin resurfacing the gold standard is ablative CO2 resurfacing.^6^ Among nonsurgical techniques for eyelid skin laxity treatment, ablative carbon dioxide (CO^2^) resurfacing has been very successful.^6^, ^7^, ^8^ However, some serious possible side effects like long‐lasting pigmentary changes, scarring, and infection caused these modalities ineligible and have caused the development of a variety of non‐ablative laser resurfacing modalities. Non‐invasive methods in aesthetic rejuvenation is rising at a notable rate.^9^, ^10^ One of the nonsurgical non‐ablative successful modalities for the eyelid skin laxity and eyebrow ptosis treatment is Endolift laser.^11^ In the present study we used Endo lift laser for excessive eyelid skin laxity and eyebrow ptosis.

### 1.1 Patients and methods

**FIGURE 1:**
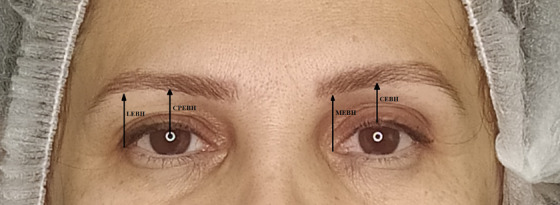
Measured indicators for eyebrow position: Eyebrow height from the center of the pupil named as (CPEBH), central eyebrow height named as (CEBH), lateral eyebrow height named as (LEBH), medial eyebrow height named as (MEBH).

Ten healthy subject (age from 35 to 60 years old) came to our department for desired treatment of eyebrow ptosis and upper eyelid laxity. The patients underwent the Endolift method. Patients with any cosmetic procedures were excluded from the study; eyelid and peeling eye inflammations, filer injection, blepharoplasty, and ptosis improvement. Informed consent was gained from the patients before the start of the treatment. Treatment was administered to the upper eyelids with Endolift™ (LASEMAR1500TM machine) was utilized at settings of power 3–5 W, T on 15 and T off 45, 3 pulse, and 300 μs pause. Before the procedure, the treatment area was carefully cleaned. A lidocaine was injected with cannula before treatment. Patients received one treatment session. The patients were evaluated 6 months after treatment. Incidence of side effects of edema, swelling, and erythema were evaluated at 1 week. Photos were taken from the patients before and 6 months after the procedure. Six months after the Endolift treatment, physicians evaluated the patient's improvement degree using the photographs by two clinical factors: skin laxity and total cosmetic result. The grading scales were as follows; 0.0 as Non, 1 as Minimal, 2 as Mild, 3 as Moderate, 4 as Advanced, and 5 as Sever. Also, the amount of improvement was analyzed as the % improvement between before and after the procedure. Also, the changes of eyebrow position were evaluated by measuring some indicators by ImagJ program before and after treatment as follow (Figure 1).

Eyebrow height from the center of the pupil is named as (CPEBH), Central eyebrow height named as (CEBH), Lateral eyebrow height named as (LEBH), and Medial eyebrow height named as (MEBH).

### 1.2 Statistical analysis

The statistical analyses were prepared via SPSS software version 12.0. Evaluations of change in lid parameters after the procedure were done with the paired *t*‐test and *p* value < 0.05 were considered statistically significant.

## 2 RESULTS

**FIGURE 2:**
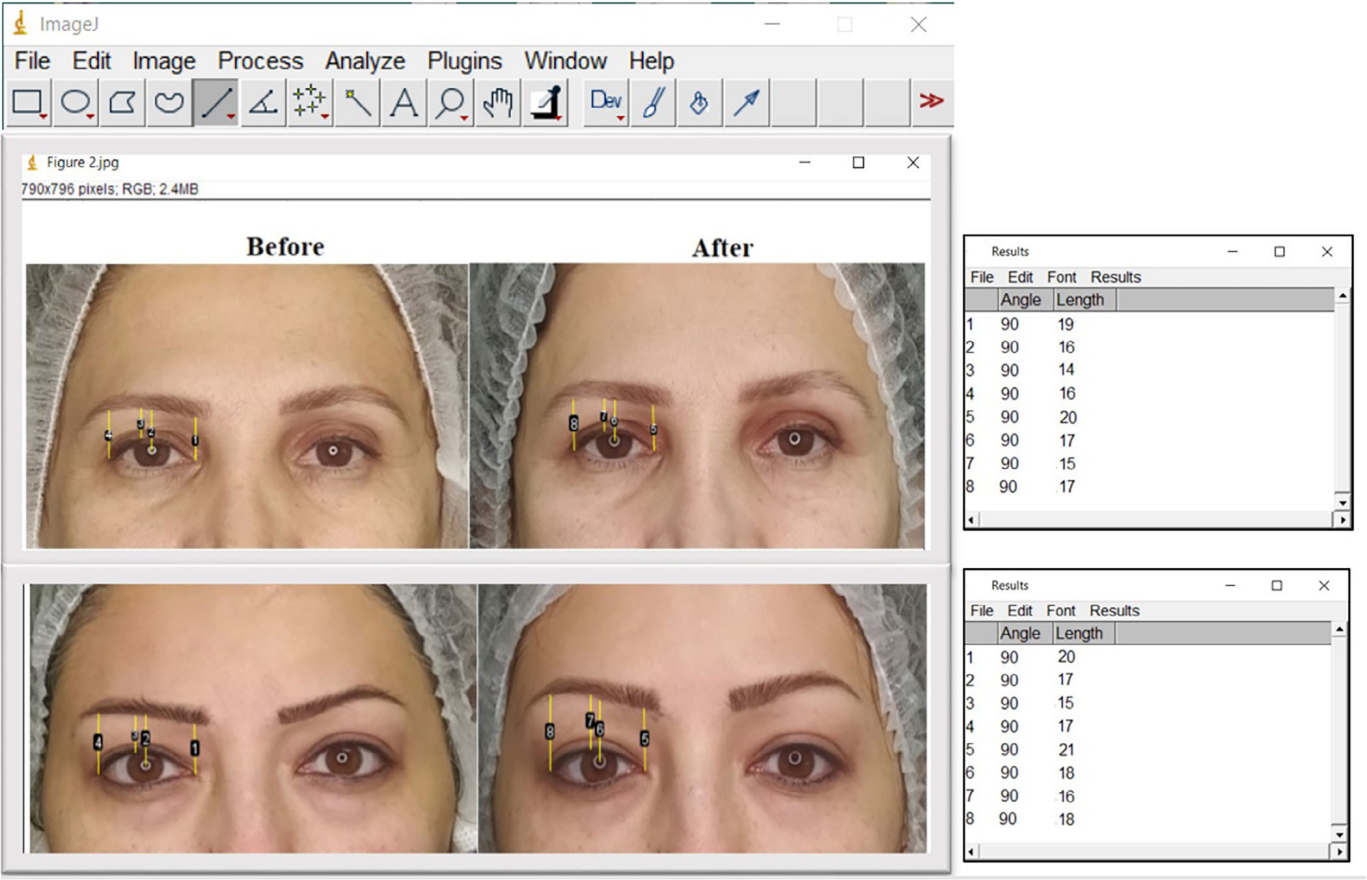
Evaluation of the patients clinical photographs using Image (before and after treatment).

A total of nine patients were received Endolift procedure. Six months post‐treatment, patients were evaluated for final clinical results. The possible side effects were evaluated at 1 week (erythema and edema). Throughout the procedure, lidocaine was injected to minimize and improve the possible pain. Two blinded physicians assessed the upper eyelid tightening improvement as a cosmetic result via photography 6 months post‐treatment. The score of skin laxity reduced from 3.5 pre‐treatment to 1.0 at 6 months post‐treatment (*p* < 0.05) with a 71.5% improvement (Table 1). The total cosmetic result score reduced from 3.5 pre‐treatment to 1.2 at 6 months post‐treatment (*p* < 0.05) for a 71.1% improvement (Table 1). Figure 2 show the clinical photographs of patients before and after treatment. After Endolift laser treatment, measurement of eyebrow heights, comprising CPEBH, CEBH, LEBH, and MEBH, were significantly higher post‐treatment compared to pre‐treatment (Table 2). All lid parameters improved significantly after Endolift laser treatment: Before treatment, the mean of CPEBH, CEBH, LEBH, and MEBH were 16.11, 14.08, 16.15, 19.13 mm and after treatment CPEBH, CEBH, LEBH, and MEBH increased 1.40, 1.49, 1.37, and 1.15 mm, respectively (Table 2). Patients reported no side effects. This treatment technique does not need general anesthesia and recovery time.

**TABLE 1:** Quantitative patient improvement in skin laxity, and total cosmetic result.

Mean scores for all patients	Skin laxity	Total cosmetic result	*p* value Pre‐treatment vs. Post‐treatment score
Pre‐treatment	3.5	3.5	<0.05
Post‐treatment	1	1.2	
Mean improvement	71.5	71.1	

**TABLE 2:** Change in lid parameters 6 months after Endolift laser treatment.

	CPEBH	CEBH	LEBH	MEBH
Before treatment	16.11	14.08	16.15	19.13
After treatment	17.51^*^	15.57^*^	17.52^*^	20.28^*^

* *p* < 0.05.

## 3 DISCUSSION

Our results showed that Endolift laser can significantly improve eyelids skin laxity and tightening. The ablative laser devices like CO2 and erbium‐doped:yttrium‐aluminumgarnet (Er:YAG) were applied for resurfacing of eyelid. These ablative laser treatments were related to some side effects like hyperpigmentation, erythema, prolonged swelling, and crusting. Furthermore, this treatment may cause severe long‐term risks such as, scarring, permanent dyspigmentation, and other textural abnormalities.^6^, ^8^, ^12^, ^13^ Fitzpatrick et al. compared the effectiveness of ablative CO_2_ versus Er:YAG on tissue tightening. The results were almost identical with both techniques. The important dissimilarity was the scarring that was saw in the Er:YAG treated.^12^ Some studies has been introduced Radiofrequency (RF) as a novel technique that can improve skin laxity with contraction that caused by heat.^7^, ^13^, ^14^, ^15^, ^16^, ^17^ RF is revealed that can lift the eyebrow, increase eyelid tightening, and contract the lower face and cheeks, and tight off the face.^7^, ^13^, ^14^, ^15^, ^16^, ^17^ In the Biesman et al. study the usage of RF was evaluated for the treatment of eyelids and their results showed 88% improvement in upper eyelid tightening and about 74% improvement in lower eyelid.^17^ Ruiz‐Esparza et al. reported the RF is effective for laxity of the lower eyelids.^15^ In a pilot study, Carruthers et al. determined that RF is effective and safe for upper and lower eyelid improvement and tightening with mild to moderate efficacy.^16^ However, RF method is laborious, slow and it takes a long time (about up to 1 h for a single eyelids treatment session). In the present study the effect of the Endolift laser was evaluated on upper eyelid laxity improvement and the outcomes presented that Endolift procedure which is a non‐ablative laser can improve the upper eyelid tightening. Also, our results showed that eyebrow position change and eyebrow heights increase significantly after treatment. Some studies have investigated the position of the eyebrow following blepharoplasty. In the Frankel et al. study they compared the eyebrow position in two groups; the group after upper blepharoplasty and the control group; their results showed that there was not significant difference in eyebrow height in the treated and control groups.^18^ In Starck et al. study, photographic analysis of patients with bilateral upper blepharoplasty displayed that eyebrow location and upper eyelid height were not significantly changed after blepharoplasty.^19^ In another study in patients with eyebrow ptosis, eyebrow position was analyzed after blepharoplasty and they reported an insignificant change in eyebrow position.^20^ Balzani et al. used fractional ultrapulse CO_2_ laser for upper eyelid dermatochalasis treatment and periorbital rejuvenation.^21^ Their results demonstrate that fractional ultrapulse CO_2_ laser is not only safe and effective for the treatment of upper eyelid dermatochalasis but also can have a nonsurgical eyebrow lift effect.^21^ In the present study we used ImagJ for eyebrow changes after Endolift laser treatment. In the Zheng et al. study the eyelid and eyebrow position were evaluated after CO_2_ Laser‐assisted blepharoptosis surgery by digital analysis. They used the digital image before and after the surgery. They analyzed the images with the ImageJ. They proved that digital image analysis is valuable in measuring the eyebrow and eyelid modifications after ptosis surgery.^22^ Using the ImageJ software for analyzing the images has some advantages. The analysis is easy and detailed, the images can be analyzed in less than 5 min and the images and information can be saved forever in the PC.

## 4 CONCLUSION

The periorbital area is one of the most sensitive areas in the face, and many techniques are used to change the eyelid laxity and position of the eyebrows. Recently the use of noninvasive or minimally invasive techniques is developed. Our results showed that Endolift laser method is an effective non‐ablative technique for upper eyelid tightening and can increase the eyebrow height. This technique does not need general anesthesia and recovery time.

## CONFLICT OF INTEREST STATEMENT

The authors declare no conflicts of interest.

## STUDY LIMITATION

Further studies are needed to assess more numbers of patients.

## Data Availability

The data that support the findings of this study are available from the corresponding author upon reasonable request.
